# A prospective investigation into the clinical impact of 4D-PET/CT in the characterisation of solitary pulmonary nodules

**DOI:** 10.1186/1470-7330-14-24

**Published:** 2014-06-05

**Authors:** Jason Callahan, Tomas Kron, Michal E Schneider, Rodney J Hicks

**Affiliations:** 1 Peter MacCallum Cancer Centre, Centre for Molecular Imaging, St Andrews Place, East Melbourne, Victoria, Australia; 2 Sir Peter MacCallum Department of Oncology, The University of Melbourne, St Andrews Place, East Melbourne, Victoria, Australia; 3 Peter MacCallum Cancer Centre, Department of Physical Sciences, St Andrews Place, East Melbourne, Victoria, Australia; 4 Department of Medical Imaging and Radiation Science, Monash University, Wellington Rd, Clayton, Victoria, Australia

## Abstract

**Background:**

While the effects of respiratory motion on measuring metabolic signal in PET/CT scanning are well known, it is still standard practice in most centres to scan patients while breathing freely with no correction for the effects of respiratory motion. The aim of this study was to investigate the impact of 4D-PET/CT in classifying lesions in patients with a radiologically-indeterminate solitary pulmonary nodule.

**Methods:**

Twenty consecutive patients with a solitary pulmonary nodule for investigation were prospectively recruited and completed a whole-body (WB)-PET/CT and 4D-PET/CT in the same session. The reporting physician initially classified the nodule using a 5-point scale (Definitely Malignant, Probably Malignant, Indeterminate, Probably benign, Definitely Benign) on the WB-PET/CT. The physician was then shown the 4D-PET/CT and asked if they would re-classify the lesion. Frequency, sensitivity, specificity and accuracy values were calculated for WB-PET/CT alone and then with the addition of the 4D-PET/CT.

**Results:**

There were no changes in the classification for nodules initially classed as either benign or malignant with the addition of a 4D-PET/CT. However changes were observed between WB and 4D-PET/CT scans in lesions initially classified as indeterminate. When indeterminate lesions were defined as malignant there was a minor increase in sensitivity (from 73% to 75%), in specificity (56%-63%) and in accuracy (65%-70%) but these results do not reach statistical significance. When the Indeterminate lesions were defined as benign there was an increase in sensitivity (from 55% to 67%) but there was a reduction in the specificity (100%-75%) and accuracy (75%-70%) with the addition of the 4D-PET/CT but again the results did not reach statistical significance.

**Conclusion:**

The addition of 4D-PET/CT is most likely to have an impact on those nodules initially classified as indeterminate on standard WB-PET/CT. In lesions classified as benign or malignant on standard WB-PET/CT the addition of a 4D-PET/CT is less likely to impact lesion classification. While 4D-PET/CT does improve the measurement of the metabolic signal, it does not overcome inherent limitations of FDG in differentiating a malignant lesion from inflammatory processes, correct for partial volume effects or compensate for the low intrinsic FDG-avidity of some malignancies.

## Background

Solitary pulmonary nodules found incidentally by anatomical imaging modalities such as computerised tomography (CT) can present a challenging diagnostic problem. The use of fluorodeoxyglucose (FDG) positron emission tomography/computerised tomography (PET/CT) to characterise a pulmonary nodule with a low to moderate pre-test probability of malignancy is now a well-established diagnostic paradigm [1]. A meta-analysis of the use of PET/CT to correctly classifying solitary pulmonary nodules found that PET had a sensitivity of 88-96% and specificity of 70-90% for malignant nodules [2]. However, despite its high reported sensitivity and specificity one factor that can confound the interpretation of a PET/CT scan is the effects of respiratory motion, particularly when the lesion is small or close to the diaphragm [3].

The aim of using FDG-PET/CT is to measure the metabolic signal in a lung nodule to differentiate benign from malignant disease and this technique has been in clinical use for over two decades [4]. However, a lesion that is moving due to respiration can have significant mis-registration between PET and CT scan and an underestimation of the true lesion metabolic signal because of blurring and inappropriate correction for the effects of soft tissue attenuation [5-7]. If a malignant lesion in not correctly classified it may lead to a delay in the appropriate management of this lesion. A method to correct the effects of respiratory motion in PET/CT is respiratory gated or 4D-PET/CT [8]. This technique entails the recording of the patients breathing pattern during the acquisition of the scan, followed by retrospectively creating a cine image of the patient breathing which removes the blurring effects of respiration.

While the effects of respiratory motion on measuring metabolic signal in PET/CT scanning are well known, it is still standard practice in most centres to scan patients while breathing freely with no correction for the effects of respiratory motion. This is mostly due to the increased complexity of acquiring a 4D PET/CT scan as well as additional equipment cost and scan time. There have been a number of studies describing improved co-registration and quantitation in lung lesions with 4D-PET/CT [7,9,10]. However, there has been much less information about the impact of 4D-PET/CT in the characterisation of pulmonary nodules as either benign or malignant. The most comprehensive paper looking at this question was a retrospective multi- centre study of 155 patients demonstrating that 4D-PET improved the overall accuracy in correctly classifying lesions from 85.7% to 92.8% [11]. One weakness of this study was the relative heterogeneity of the data in terms of technical specification of the various cameras, uptake times and acquisition parameters at the six centres involved in the study. Another study published in 2010 by Vicente *et al.* investigated the impact of 4D-PET/CT in 42 lung lesions with a very low FDG signal initially thought to be benign. They found a change in classification from benign to malignant in 17/42 (40%) cases [12]. To date there is a lack of prospective data in the literature investigating the impact of using 4D-PET/CT in all patients with a radiologically-indeterminate SPN.

Hence, the aim of this study was to investigate the impact of 4D-PET/CT in the characterisation of solitary pulmonary nodules in a routine clinical environment, using a homogenous acquisition and automated processing technique with a current-generation, time-of-flight PET/CT device.

## Methods

### Patient population

Twenty consecutive patients were prospectively recruited between November 2011 and July 2012. The study was approved by the Peter MacCallum Clinical Governance and Ethics Committee, and all patients provided written informed consent. All patients recruited had been referred to the Peter MacCallum Centre for molecular imaging to have a FDG-PET/CT to investigate a newly detected solitary pulmonary nodule on CT. Patients were included if they had a lesion of at least 10 mm in size that was either deemed of indeterminate nature based on CT appearances or deemed not to be amenable to biopsy despite being considered suspicious of malignancy. A patient’s pre-test risk of malignancy was not an inclusion criterion. Patients were included regardless of their smoking status or any prior malignancy.

### Patient preparation

After a fasting time of at least six hours, patients were administered with an FDG dose of 3.2 MBq/kg. Patients were instructed to lie quietly in an uptake room for at least 60 minutes. All patients were scanned on a GE-Discovery690 (GE Medical Systems Milwaukee, WI) with a mean uptake time of 66 min (range 60-75 mins). Patients were positioned supine with their arms up and the infrared reflective marker block for the Varian RPM respiratory tracking system (Varian Medical Systems, Palo Alto, CA) placed on their abdomen. A standard whole body PET/CT (WB-PET) scan was completed and then immediately followed by a 4D-PET/CT centred over the pulmonary nodule of interest.

### Whole Body PET/CT acquisition and processing

Patients were instructed to breathe freely throughout the whole body PET/CT scan. A CT scan extending from the base of brain to the proximal thighs was acquired with the following exposure parameters; 140kv, Smart mA range 40–150, rotation time 0.5 sec, pitch 0.984. This was immediately followed by a whole body-PET scan with the following acquisition parameters; 3D frame mode, 192 matrix, 2.5 mins/step, 11slice overlap. The WB-PET was then reconstructed using OSEM iterative reconstruction with 2 iteration, 18 subsets, 5 mm Gaussian filter, time of flight and SharpIR.

### 4D-PET/CT acquisition and processing

Immediately following the completion of the WB-PET the patient moved into position to begin the 4D-CT scan. After moving the patient into position the breathing was allowed to stabilise and recorded using the Varian Real-Time Patient Monitoring (RPM) system. The Varian RPM system uses an infrared camera to track the motion of a box placed on the patient’s abdomen. Once the breathing had stabilised, a step and shoot 4D-CT scan was performed using the following parameters: Cine CT, 10 mA, 140 kV, Cine duration = breathing period + 1.5secs, and the cine time between images = breathing period/10. After completion of the 4D-CT scan, the respiratory trace was saved and a new trace was established to use for the 4D-PET.

Immediately after the 4D-CT was completed the patient was moved into the PET position and the breathing was allowed to stabilise again before beginning the PET acquisition. This was acquired as a 3D list mode acquisition while recoding the patient’s breathing trace. The 4D-PET was acquired for 10 minutes.

The GE automatic phase matching software was used to process the 4D-PET/CT scan. The 4D-CT was retrospectively binned into five frames using the saved respiratory trace. The acquired 3D-List mode PET scan was retrospectively binned into five frames and each phase of the 4D-PET was automatically corrected for attenuation with the respective phase of the 4D-CT. The 4D-PET was reconstructed using the same reconstruction parameters as the WB-PET.

### Characterisation of lesions

A five point scale as outlined by Fletcher et. al. [13] was used to classify the nodules. Fletcher’s five point criteria are outlined in Table 1.

**Table 1:** Fletcher’s PET and CT criteria for rating SPN

**Category**	**Relationship between SPN18F-FDG uptake and likelihood of malignancy**	**Relationship between CT SPN characteristics and likelihood of malignancy**
**Definitely benign**	No increased uptake—uptake essentially the same as in reference lung tissue (generally corresponds to an SUV of 0.6–0.8)	Central laminated or diffuse calcification
		Popcorn pattern of calcification
		Lesion with cavitations and wall thickness < 1 mm
**Probably benign**	Uptake substantially less than in blood pool (general mediastinal activity) but greater than in reference lung tissue (SUV greater than 0.6–0.8 but less than 1.5–2.0)	Large (< ;2 cm) dominant nodule with satellite lesions
		Solid nodule with polygonal shape or smooth and well-defined margin
		Diameter < 10 mm; lobulated margin contours
**Indeterminate**	Uptake 2–3 times that in reference lung tissue but less than in blood pool (generally corresponds to SUV of 1.5–2.0 but less than 2.5)	All other characteristics not defined in other likelihood categories
**Probably Malignant**	Uptake greater than in blood pool (blood pool generally corresponds to an SUV of 2.5)	Diameter > 2 cm
		Ground-glass opacity with round shape
		Mixed ground-glass opacity with central zone of high attenuation
**Definitely Malignant**	Uptake much greater than in blood pool—anything substantially greater than SUV of 2.5	Densely spiculated margin, ragged margin Lesion with cavitations and wall thickness > 16 mm

The scans were reported as a single read where the physician was first shown the WB-PET/CT scan and asked to characterize the pulmonary nodule using the five catagories. The same reporting physician was then shown the 4D-PET/CT scan and asked if the additional information changed the classification of the pulmonary nodule. In order to replicate our usual reporting process, the reporting clinician was able to review all relevant clinical information and any prior imaging results. The reporting physician also incorporated the morphologic appearances of the nodules into account when classifying nodules into the five categories as per standard clinical practice. For example a solid nodule with smooth well-defined margins and no uptake was likely to be benign whereas a lesion with high uptake and a densely spiculated margin is likely to be malignant. If based on all the available information the nodule could not be confidently categorised as either benign or malignant they were categorised as indeterminate. For indeterminate nodules on CT, the PET scan was given a stronger weighting than the CT appearances but for lesions unsuitable for biopsy, both modalities were considered in arriving at a final diagnosis. Accordingly, the initial diagnosis was risk-adapted and only the incremental diagnostic information of the gated study was assessed. The 20 scans were reported by four imaging specialists who each had a minimum of 10 years clinical reporting experience.

### Patient follow-up

Patients were followed up until a definitive diagnosis was obtained. Follow up was obtained for all 20 patients and compared to the WB-PET and 4D-PET diagnosis. If the nodule was resected, histopathology was used to confirm the diagnosis. Any lesions that resolved on subsequent imaging without oncological intervention were considered of a benign origin.

### Statistical analysis

Frequencies were calculated for the five classification types to compare the frequencies of classification in WB-PET/CT and 4D-PET/CT scanning. The sensitivity, specificity and accuracy with confidence intervals were also calculated to compare the findings of the two scanning techniques. As an indeterminate lesion is neither benign of malignant we have calculated the accuracy with indeterminate lesions classified as either being all malignant or all benign. This is the same method employed by Guerra et. al. to look at the added diagnostic value of 4D-PET/CT [11]. A McNemars test was used to detect any difference in the proportion of lesions classified as indeterminate on WB-PET/CT compared to 4D-PET/CT.

## Results

### Lesion diagnosis

**Figure 1:**
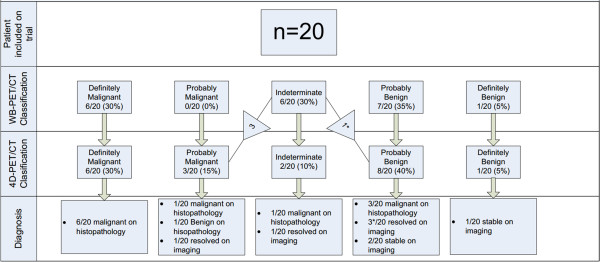
Classification of solitary pulmonary nodules observed on WB-PET/CT and 4D-PET/CT scans and subsequent diagnosis (Total N lesions = 20).

As outlined in Figure 1, 13 patients had a diagnosis confirmed by histopathology, 4 resolved on imaging (mean 6 months, 3.7-11.6 months) and 3 were stable more than 12 months after the PET scan (mean 14 months, 12–18 months). Of note are three patients that were classified a probably benign on imaging but showed an interval increase in size at an early imaging follow-up. These were subsequently confirmed as malignant on histopathology without and interval change in stage. Thus, only 2 patients underwent biopsy for a non-malignant process and no patients were inappropriately observed.

### Impact of 4D-PET/CT in lesion classification

The proportion of patients in each category and their subsequent diagnosis are outlined in Figure 1. In this cohort, there were no changes in the PET classification of nodules initially classed as either benign or malignant on standard WB PET/CT. The only changes in classification between WB and 4D-PET/CT scans were in lesions initially classified as indeterminate.

### Lesions initially classified as Benign or Malignant on WB-PET/CT

Fourteen out of the 20 patients were classified as either benign (8/20 (40%)) or malignant (6/20, (30%)) based on the WB-PET/CT result. Of these fourteen cases there was no change in lesion classification with the addition of the 4D-PET/CT. All six cases classed as malignant, we subsequently confirmed true positive. Three out of the eight lesions classified as benign on FDG-PET were subsequently found to be malignant on follow up indicating they were incorrectly classified on both WB and 4D-PET/CT scans. One of the three incorrectly classified lesions was a carcinoid, while the other two were small adenocarcinomas.

### Lesions initially classified as indeterminate on WB-PET/CT

**Figure 2:**
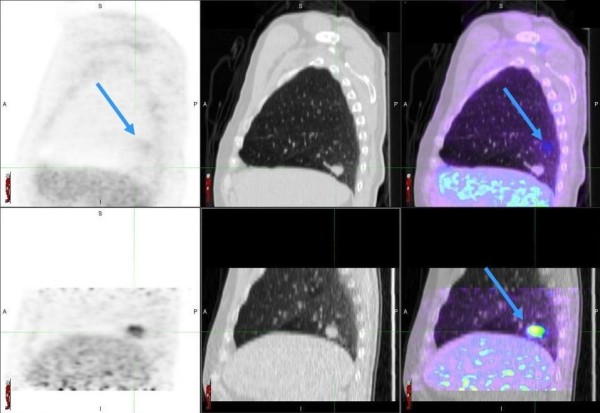
**Lesion in the Right Lower Lobe was initially indeterminate based on the WB-PET/CT scan (top row - SUVmax = 1.8).** The 4D-PET/CT scan (bottom row –SUVmax = 4.8) revealed FDG uptake in the lesion influencing the reporting physician to re-classify the lesion as probably malignant. The blue arrow in the top left hand cell shows how the significant mis-registration between the PET and CT scans is then well corrected on the 4D-PET/CT in the bottom right hand panel.

**Figure 3:**
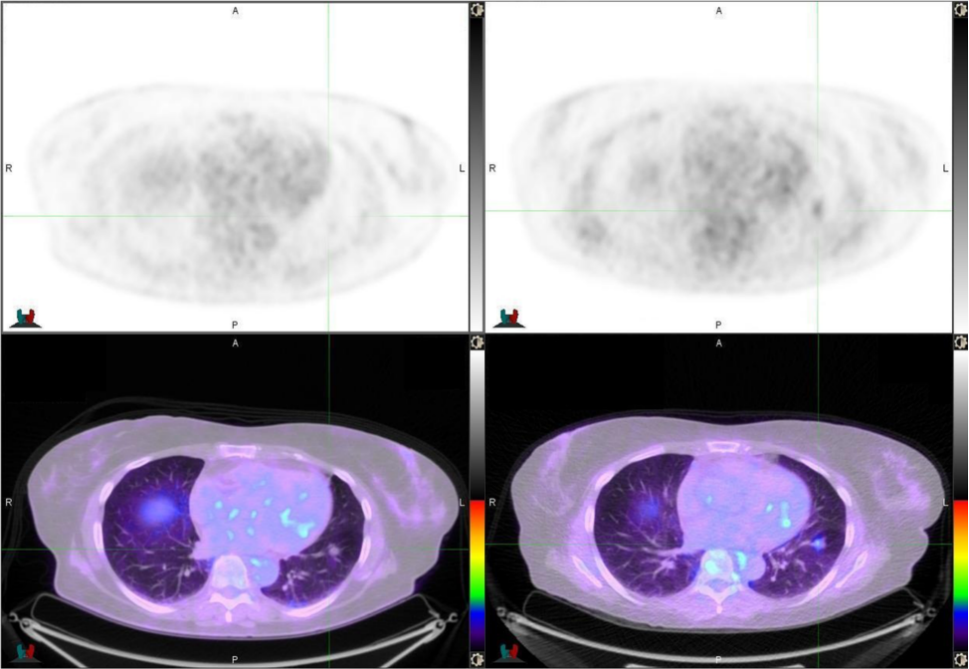
**Lesion in the left lower lobe that was original classified on the WB-PET/CT (left column) as indeterminate and was subsequently changed to probably malignant on the 4D-PET/CT (right column).** The SUVmax increased from 1.4 to 2.0. This lesion was subsequently confirmed as an adenocarcinoma.

Six of the 20 lesions (30%) were initially classified as indeterminate on the WB-PET/CT scan. The results for these six lesions are outlined in Table 2. In four out of these six lesions, the 4D-PET/CT influenced the reporting physician to change their classification. However the reduction in the proportion of indeterminate lesions from 6/20 to 2/20 was not statistically different (p = 0.1336).In one out of these four cases, the continued absence of metabolic signal on the 4D-PET/CT in a lower lobe lesion influenced the physician to classify the lesion as probably benign, which was found to be correct. In three out of the four cases higher FDG signal was detected (SUVmax increase: 1.8-4.8 [167% increase], 1.4-2.0 [43%] and 1.8-2.5 [34%], respectively) on the 4D-PET/CT scan leading the reporting physician to classify the lesion as more likely to be malignant. Two of three of these lesions were subsequently found to be benign. Figure 2 shows an example of a 2.6 cm lesion that was initially indeterminate but changed to probably malignant based on the additional signal detected on the 4D-PET/CT. This lesion was subsequently resected and found to be a granulomatous lesion. Figure 3 shows a case where the higher SUVmax on the 4D-PET/CT influenced the reporting physician to reclassify the lesion from indeterminate to probably malignant. This was subsequently resected and found to be malignant.

**Table 2:** This table shows the progression of lesions initially categorised as indeterminate on Whole Body PET/CT, any change in classification with the additional 4D-PET/CT and in lesions final diagnosis

**WB-PET/CT finding**	**4D-PET/CT finding**	**Final diagnosis**	**Method of diagnosis**
Indeterminate	Indeterminate	BENIGN	Resolution on imaging
Indeterminate	Indeterminate	MALIGNANT	Lesion Resected - Adenocarcinoma
Indeterminate	Probably Benign	BENIGN	Resolution on imaging
Indeterminate	Probably Malignant	BENIGN	Lesion Resected - Granulomatous
Indeterminate	Probably Malignant	BENIGN	Resolution on imaging
Indeterminate	Probably Malignant	MALIGNANT	Lesion Resected - Carcinoid

Table 3 shows the relative sensitivity, specificity and overall accuracy for in initial classification on the WB-PET/CT alone and then with the addition of the 4D-PET/CT. If all indeterminate lesions were defined as malignant irrespective of acquisition method, the 4D-PET/CT scan had a sensitivity, specificity and accuracy the WB PET/CT but these results did not reach statistical significance. However, when the indeterminate lesions were defined as benign, the accuracy and specificity of 4D-PET/CT was lower than WB PET/CT with the addition of the due to two additional false positive findings that had been classified as benign on the WB-PET/CT scan but again the results did not reach statistical significance (Table 3).

**Table 3:** Shows the relative sensitivity, specificity and overall accuracy with 95% confidence intervals when indeterminate lesions were classified as either being Malignant (left column) or Benign (right column)

	**Indeterminate as malignant**		**Indeterminate as benign**	
	**WB-PET/CT**	**4D-PET CT**	**WB-PET/CT**	**4D-PET CT**
SENSITIVITY	73% (39-93%)	69% (39-91%)	55% (23-83%)	67% (35-90%)
SPECIFICITY	56% (21-86%)	71% (29-96%)	100% (66-100%)	75% (35-97%)
Accuracy	65% (35-87%)	70% (39-90%)	75% (51-91%)	70% (39-90%)

None of the observed difference were statistically significant.

## Discussion

At our facility, the use of PET/CT to classify solitary pulmonary nodules is restricted to lesions that are either indeterminate on CT criteria or, if suspected to be malignant, considered to be unsuitable for percutaneous biopsy. In the latter situation, FDG PET/CT is used both for planning the best technique and site for biopsy [14] and for staging in the event that malignancy is confirmed. Accordingly, there is a significant pre-test selection bias in this prospective series. However, we believe this to be an appropriate clinical setting in which to evaluate the incremental value of respiratory-gated (4D) PET/CT.

We have found that when the reporting physician is able to classify a lesion as either benign or malignant on conventional WB PET/CT, an additional 4D-PET/CT did not change their diagnosis. The only clinical situation where the 4D-PET/CT was found to impact the reporting physician’s classification was when the initial finding on the WB-PET/CT was considered to be indeterminate. In this study, the rate of indeterminate findings reduced from 30% to 10% with the addition of 4D-PET/CT. This is consistent with the work by Guerra and colleagues who noted that the main discrepancy between WB-PET/CT and 4D-PET/CT was in indeterminate findings which were reduced from 24.3% to 4.4% with the addition of a 4D-PET/CT [11].In the four indeterminate lesions that had a change in classification from indeterminate to probably malignant, two were found to be benign. It is clear that mitigating the effects of the respiratory motion artefact to provide a more representative estimation of true FDG-avidity does not change the inherent weakness of FDG in differentiating between a malignant lesion and an inflammatory process (Figures 2 and 3). Additionally, respiratory gating can’t overcome the issues posed by partial volume effects or improve the sensitivity of PET in the presence of low FDG-avidity, as seen in some low-grade malignant nodules including carcinoid tumours.

The results of this prospective cohort is also similar to that presented by Vicente et al. [12] who showed that in lesions with low FDG avidity (N = 42) the addition of 4D-PET/CT decreased specificity (100% to 72%) due to additional false positive findings. However, there was an increase in the overall accuracy (from 45% to 62%), due improved sensitivity. As has been shown in a number of studies, the 4D-PET/CT scanning technique removes the respiratory motion artefact and reveals the true metabolic signal [7-9]. While an improvement in the sensitivity of detection of malignancy may be desirable, even at the risk of additional false positives, most tumours with low FDG-avidity tend to have a less aggressive natural history and it may be reasonable to observe these for growth on CT rather than subjecting patients to the morbidity that can be associated with pathological characterisation.

It has been proposed that dual time point imaging may be useful in differentiating benign from malignant lung nodules [15]. However, a meta analysis did not find that this technique improved the overall accuracy of FDG-PET for this indication [15]. This may be because the lung nodules at both time points are still affected by respiratory blurring. The dual time point method assumes an increase in SUV uptake is indicative of malignancy but one needs to consider that the measurement of SUV in moving lesions is less accurate [5,9,11,12]**.** An additional confounding factor for lesions subject to the respiratory motion in standard WB-PET/CT is inaccurate attenuation correction due to mis-registration. The dual time point method does not address any of these confounding factors. In this study, the 4D PET/CT was performed after the WB PET/CT and it is therefore possible that some of the increased sensitivity observed related to delayed imaging.

The routine addition of a 4D-PET/CT using this acquisition protocol is unlikely to be feasible for all patients in a busy clinical PET centre. The described 4D-PET/CT scanning technique requires additional scan time, infrastructure and patient co-operation. There is also an increase in radiation dose due to the 4D-CT scan. This is a particularly important consideration in the patient cohort who may not have any malignancy. Therefore, it is important to determine when to apply this scanning technique to improve the overall accuracy of FDG-PET. The results of this and other studies study suggest that the most efficient use of 4D-PET/CT is in those pulmonary nodules that are of an indeterminate nature based on the standard WB-PET/CT technique. This process requires the reporting physician to review the WB-PET/CT prior to the patient leaving the imaging centre. While this can be disruptive for those patients completing a 4D-PET/CT it will avoid a lot on unnecessary 4D scanning for lesions that are unlikely to have their classification impacted by a 4D scan.

A limitation of the data presented is the relatively small number of patients. However, given that the results are similar to the larger retrospective cohort of Guerra *et al.,*[11] we believe that these prospective findings strengthen the overall body of evidence demonstrating that 4D-PET/CT is best applied in indeterminate pulmonary lesions.

In this paper we have also not used a SUVmax cut off for the classification of malignancy because any cut off value is arbitrary and certainly an optimal diagnostic threshold has not been defined for modern time-of-flight scanners or using respiratory gating to date. The lesion shown in Figure 3 had a SUVmax of 4.8 but was subsequently found to be granulomatous. While this lesion was incorrectly classified as malignant, in reality the clinical implication of this result is that malignancy should be excluded by early pathological characterisation if possible. Also, as shown in a recent paper by Lee et. al. [16], the degree of FDG uptake is dependant on the tumour type meaning that an arbitrary cut off value may incorrectly classify lower grade tumours. It is for these reasons that an arbitrary SUVmax cut-off value without taking into account the patient’s likelihood for malignancy is likely to be less accurate.

With the widespread use of CT to detect solitary pulmonary nodules there will be an increasing need to use PET to characterise indeterminate lesions found by this imaging modality [17]. Adeonocarcinoma is now the most common lung malignancy, which will challenge the ability of FDG-PET to correctly classify lesions due to the variability in glucose uptake across the different type of adenocarcinomas [16]. An additional 4D-PET cannot overcome false positive or negatives due to tracer kinetics, but there may be an impact on management if glucose uptake sufficiently predicts mitotic behaviour allowing allocation to a CT observational strategy without concern of interval stage migration. A 4D acquisition may be a tool to improve the overall accuracy of FDG-PET in this setting.

## Conclusion

The addition of 4D-PET/CT is most likely to have an impact on those nodules initially classified as indeterminate on standard WB-PET/CT. In lesions classified as benign or malignant on standard WB-PET/CT the addition of a 4D-PET/CT is less likely to impact lesion classification. While 4D-PET/CT does improve the measurement of the metabolic signal, it does not overcome inherent limitations of FDG in differentiating a malignant lesion from inflammatory processes, correct for partial volume effects or compensate for the low intrinsic FDG-avidity of some malignancies.

## Competing interests

The authors declare that they have no competing interests.

## Authors’ contribution

JC carried out the design of study, recruiting of patients, acquisition of studies, follow-up of patients, analysis of data and writing of manuscript. MS, TK and RH contributed to design of study and analysis of results and writing of manuscript. All authors read and approved the final manuscript.
